# Microbiological characteristics of the lower airway in adults with bronchiectasis: a prospective cohort study

**DOI:** 10.1186/s12931-024-02903-1

**Published:** 2024-07-17

**Authors:** Jie-lin Duan, Cai-yun Li, Ying Jiang, Chao Liu, Pan-rui Huang, Li-fen Gao, Wei-jie Guan, Lin-Ling Cheng

**Affiliations:** 1 State Key Laboratory of Respiratory Disease, National Clinical Research Center for Respiratory Disease, Department of Allergy and Clinical Immunology, Guangzhou Institute of Respiratory Health, the First Affiliated Hospital of Guangzhou Medical University, Guangzhou, P.R. China; 2 State Key Laboratory of Respiratory Disease, National Clinical Research Center for Respiratory Disease, National Center for Respiratory Medicine, Department of Respiratory and Critical Care Medicine, Guangzhou Institute of Respiratory Health, the First Affiliated Hospital of Guangzhou Medical University, Guangzhou, P.R. China; 3 Guangzhou National Laboratory, Guangzhou, China; 4 Medical Department, Hangzhou Matridx Biotechnology Co., Ltd, Hangzhou, People’s Republic of China

**Keywords:** Bronchiectasis, Metagenomic next-generation sequencing, *Pseudomonas aeruginosa*

## Abstract

**Background:**

Microbial infection and colonization are frequently associated with disease progression and poor clinical outcomes in bronchiectasis. Identification of pathogen spectrum is crucial for precision treatment at exacerbation of bronchiectasis.

**Methods:**

We conducted a prospective cohort study in patients with bronchiectasis exacerbation onset and stable state. Bronchoalveolar lavage fluid (BALF) was collected for conventional microbiological tests (CMTs) and metagenomic Next-Generation Sequencing (mNGS). Bronchiectasis patients were monitored for documenting the time to the next exacerbation during longitudinal follow-up.

**Results:**

We recruited 168 eligible participants in the exacerbation cohorts, and 38 bronchiectasis patients at stable state at longitudinal follow-up. 141 bronchiectasis patients at exacerbation onset had definite or probable pathogens via combining CMTs with mNGS reports. We identified that *Pseudomonas aeruginosa,* non-tuberculous mycobacteria*, Haemophilus influenzae, Nocardia spp,* and *Staphylococcus aureus* were the top 5 pathogens with a higher detection rate in our cohorts via combination of CMTs and mNGS analysis. We also observed strong correlations of *Pseudomonas aeruginosa, Haemophilus influenzae, non-tuberculous mycobacteria* with disease severity, including the disease duration, Bronchiectasis Severity Index, and lung function. Moreover, the adjusted pathogenic index of potential pathogenic microorganism negatively correlated (*r* = -0.7280, *p* < 0.001) with the time to the next exacerbation in bronchiectasis.

**Conclusion:**

We have revealed the pathogenic microbial spectrum in lower airways and the negative correlation of PPM colonization with the time to the next exacerbation in bronchiectasis. These results suggested that pathogens contribute to the progression of bronchiectasis.

**Supplementary Information:**

The online version contains supplementary material available at 10.1186/s12931-024-02903-1.

## Introduction

Bronchiectasis is a chronic airway disease that is characterized by progressive and irreversible dilatation of bronchus due to the recurrent damage of the bronchial wall [1]. Clinical presentations of bronchiectasis include chronic cough, purulent phlegm, hemoptysis, and repeated pulmonary infection [2, 3]. However, the pathogenesis of bronchiectasis remains elusive. The “Vicious vortex” hypothesis suggests that the changes in the airway microenvironment (such as impaired mucociliary clearance) facilitate the colonization of microorganisms and increase the future risk of infection [4, 5]. The failure to clear pathogens could, in turn, induce airway neutrophilic inflammation and progression to airway destruction [4, 5].

Recurrent infections play a central role in the pathogenesis of bronchiectasis. Previous studies have demonstrated that bronchiectasis patients with *Pseudomonas aeruginosa* infection had more severe clinical symptoms, increased exacerbation frequencies, greater disease severity, and worse lung function [6]. Although effective among bronchiectasis patients, antibiotics cannot readily eradicate a number of potential pathogenic microorganisms (PPMs). Furthermore, the colonization status and higher bacterial loads of PPMs have been linked to poorer clinical outcomes and antibiotic response in patients with bronchiectasis and COPD [7, 8].

In light of the pivotal roles of infection in driving the progression of bronchiectasis, it is crucial to identify PPMs and evaluate their abundance within the lower airways. These steps are essential for gaining insights into the pathophysiology of bronchiectasis and for guiding antibiotic treatment at onset of exacerbations. Timely and appropriate use of antibiotics is pivotal for preventing from the deterioration of pulmonary function and progression to exacerbation of bronchiectasis [9]. In principle, metagenomic next-generation sequencing (mNGS) can detect microorganisms of any known genomic sequences and has been widely used for etiological diagnosis of the respiratory tract, central nervous system, and bloodstream infections, etc. [10–12]. Moreover, mNGS can cover a broader spectrum of pathogens than conventional microbiological tests (CMTs) such as bacterial and fungal culture, serologic testing, and polymerase chain reaction (PCR), thus better informing the clinicians an appropriate antibiotic choice.

Traditionally, sputum specimens have been used for pathogen testing in bronchiectasis. However, sputum is susceptible to oral contamination and may not accurately reflect the pathogen burden in the lower (particularly distal) respiratory tract. Here, we aimed to evaluate the utility of CMTs and mNGS for detecting pathogens, and outline the characteristics of microorganisms in the lower airways in bronchiectasis. Furthermore, we investigated the association between the type or load of pathogens and prognosis in bronchiectasis.

## Methods

### Study population and samples collection

The patients were enrolled between Dec 17th 2020 and Sept 1st 2022 from the First Affiliated Hospital of Guangzhou Medical University, and followed until Oct 1st 2023 for recording the time to the next exacerbation. All patients have written informed consent. Eligible patients were aged 18 years or older, and had a high-resolution computed tomography (HRCT)-confirmed diagnosis of bronchiectasis. Exacerbations were defined as significantly increased or new-onset respiratory symptoms (chronic cough, infection, fever, purulent sputum, hemoptysis) that needed antibiotics administration as defined by the European Respiratory Society [13]. Stable state was defined as the absence of new symptoms and no changes in bronchiectasis therapy in the past four weeks [14]. Patients were excluded if they were unable to tolerate bronchoscopy. Patients with bronchiectasis underwent bronchoscopy of collection of bronchoalveolar lavage fluid (BALF) at onset of exacerbations and during the stable state. The disease severity was evaluated with the bronchiectasis severity index (BSI) and E-FACED score. Lung function was assessed by the forced expiratory volume in one second (FEV_1_) and the FEV_1_/forced vital capacity (FVC) ratio. Disease duration, the time since the first diagnosis of bronchiectasis by radiology, was recorded during consultation of case history. The diagnosis of exact infectious etiology of each patient was comprehensively made based on a composite reference standard as previous report [10], including CMTs results, mNGS reports, radiological findings, clinical presentations, therapeutic response to antibiotics, and further adjudication by an independent expert panel involving two independent clinicians.

### CMTs

The CMTs included bacterial/mycoplasma/chlamydia/fungal culture, galactomannan antigen test, (1,3)-β-D-glucan test and cryptococcal polysaccharide antigen test. Sputum and BALF were collected from each patient, transported to the microbiology laboratory, and processed (< 2 h) for culture. Sputum samples with > 25 leukocytes and < 10 squamous epithelial cells (at low-power field) were deemed eligible. Blood/chocolate agar (bioMérieux) and Sabouraud agar were used as the culture media. Multiplex PCR was used for the identification of non-tuberculous mycobacteria.

### mNGS

The internal control (IC) DNA, a double-stranded DNA fragment referred as a spike in control, was synthesized, amplified by PCR (TAKARA PrimeSTAR® HS DNA Polymerase, Cat# R044) and purified using magnetic beads (Matridx, Cat# MD012). All procedures were performed inside a Biosafety cabinet. Qubit fluorometric quantitation was performed on the amplicons (Thermo Fisher, Qubit dsDNA HS Assay Kit, Cat# Q32854). The nucleotide sequences of IC DNA have been reported previously. ICs were added to each BALF sample prior to nucleic acid extraction at a final concentration of 0.02 ng/μL. DNA sequencing library was prepared by enzymatic fragmentation, end repairing, terminal adenylation and adaptor ligation (NGSmaster™ library preparation, Matridx, Cat# MAR002) [15]. Concentration of DNA libraries was quantified by real-time PCR (KAPA) and pooled. Shotgun sequencing was carried out on illumina Nextseq™ platform. Approximately 20 million of 75 bp single-end reads were generated for each library [16, 17]. For each run, a negative control (medium containing 10^6^/mL JURKAT cells) was included for quality control.

Raw sequences were processed by a bioinformatic pipeline, which included: 1) adapter sequences and low-quality bases (Q-score cutoff of 20) were trimmed by Fastp (https://github.com/OpenGene/fastp); 2) Host reads were filtered by mapping to the human reference genome (GRCh38.p13) using Burrows Wheeler alignment (BWA, http://bio-bwa.sourceforge.net) [18]; 3) After removal of low-complexity reads, the remaining sequences were aligned by BWA to an in-house reference database (curated from NCBI nt database (ftp://ftp.ncbi.nlm.nih.gov/blast/db/) and GenBank (ftp://ftp.ncbi.nlm.nih.gov/genomes/genbank/)) [18]. We defined that reads with 90% identity of reference were mapped reads. In addition, reads with multiple locus alignments within the same genus were excluded from the secondary analysis. Only reads mapped to the genome within the same species were considered.

Microbial reads identified from a library were reported if: 1) the sequencing data passed quality control filters (library concentration > 50 pM, Q20 > 85%, Q30 > 80%); 2) negative control in the same sequencing run does not contain the species or the Reads Per Million (RPM) of the sample / RPM of the negative control ≥ 5.

### Calculation of the adjusted pathogenic index (API)

To analyze the microbial abundance in BALF, we used the adjusted pathogenic index (API) to reflect the bacterial load of microbial species as described previously [19]. Briefly, for each library, we chose the reads per million (RPM) mapped reads as the normalization method for mNGS reads as previous report [20]. RPM was calculated using the formula: gene reads / the total mapped reads (millions). We added spike (IC DNA) to each BALF sample and carried out mNGS testing to evaluate the relationship between spike RPM and nucleated cell count. We observed that spike RPM was inversely correlated with cell count (R^2^ = 0.6278), suggesting that cell count in BALF samples correlated to increased human DNA contamination in high cell count samples, and hence reduced spike in recovery. The RPM of the internal control was defined as SRPM (spike reads per million mapped reads).

The API was calculated according to the following formula: API = log_2_[(1,887,800 × RPM / SRPM) + 1] × 1000. Specifically, the factor 1,887,800 originated from 1.8878 μg × 1,000,000. 1.8878 μg represents the amount of spike nucleic acid added per liter of BALF, and 1,000,000 is a coefficient to normalize the ratio of (1,887,800 × RPM / SRPM) similar to 1. The API score is logarithmically transformed to ensure a 5-digit range.

### Association between PPM colonization and clinical outcomes in bronchiectasis

The patients at clinical stable state were regularly followed-up to record the time to next exacerbation. According to microbial results from mNGS and CMTs, bronchiectasis patients at clinical stable state were clarified into PPM and non-PPM group according to the presence or absence of PPMs as reported previously [21]. Briefly, PPMs are those microorganisms that are recognized for causing respiratory infections, like *Pseudomonas aeruginosa*, *Enterobacteriaceae*, *Haemophilus influenzae*, *Staphylococcus aureus*, *Streptococcus pneumoniae*, and *Moraxella catarrhalis*, regardless of their presence in other body areas. Conversely, non-PPMs, like *Streptococcus viridans* group, *Neisseria species*, *Corynebacterium species*, and *Candida species*, are typically found in the oropharynx or gut and not associated with respiratory infections in healthy individuals [21]. Then, the correlation of API with the time to the next exacerbation in PPMs or non-PPMs group was analyzed.

### Statistical analysis

The SPSS 25.0 (IBM Corporation, USA) package was used for statistical analysis. The quantitative variables were evaluated as mean (standard deviation, SD) or normal distribution or as median (inter-quartile range, IQR) for non-normal distribution. The qualitative or dichotomized variables were evaluated as count (percentage of the total). Associations for the between-group differences were tested using the Kruskal–Wallis or the Wilcoxon matched paired signed-rank test. Pearson’s correlation analysis was used to examine the associations of API in PPM or non-PPM group with the time to next exacerbation.

## Results

### Study profile and patient characteristics

**Fig. 1:**
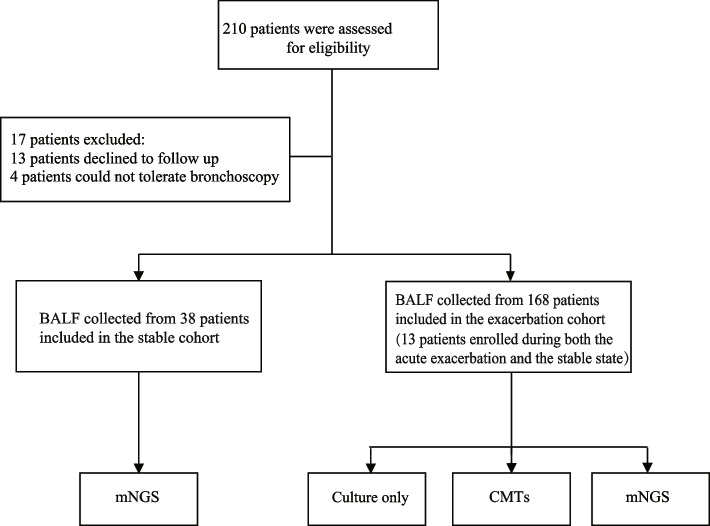
The design of the study was illustrated in the flow chart

Between Dec 17th 2020 and Sept 1st 2022, 168 eligible participants (excluding 13 patients who declined follow-up and 4 patients who could not tolerate bronchoscopy) were recruited into the exacerbation cohorts. Meanwhile, 38 bronchiectasis patients (including 13 patients who were enrolled during both the acute exacerbation and the stable state) in stable state were recruited, and were regularly followed-up to monitor the time to the next exacerbation. All participants provided sputum and BALF sample for CMTs as described previously (Fig. 1 and Supplementary Table 1). mNGS was applied for BALF samples only. The exacerbation patients had an average age of 54 (44, 64) years, with a mean disease duration of 10 (3, 20) years. The most common symptoms at exacerbation onset were increased cough and sputum (62/168, 36.9%). The mean FEV_1_ and FEV_1_/FVC of exacerbation bronchiectasis was 1.59 (1.03, 2.07) liters and 72.13% (59.01%,81.71%), respectively. The BSI and E-FACED score was 10.0 (7,11.75) and 3.0 (1, 4), respectively. 9.5% of patients were cigarette smokers and 17.9% had a previous history of pulmonary tuberculosis (30/168, 17.9%) (Table 1).

**Table 1:** Demographic and clinical characteristics of 193 patients

Clinical characteristics^a^	Exacerbation cohort (*n* = 168)	Stable cohort (*n* = 38)	*P*-value
Age	54(44, 64)	45.5 (34.75,63.25)	0.042
Male	46 (27.4%)	14 (36.84%)	0.246
BMI	20.42(18.56,22.83)	21.23 (19.72,25.44)	0.027
Disease duration (Years)	10 (3,20)	8.5 (5.75,12.25)	0.769
Number of lobes affected			0.010
1	19 (11.3%)	5 (13.2%)	
2	31 (18.5%)	7 (18.4%)	
3	99 (58.9)	14 (36.8%)	
4	16 (9.5%)	8 (21.1%)	
5	3 (1.8%)	4 (10.5%)	
FEV_1_ (L)	1.59 (1.13,2.07)	1.86 (1.22,2.35)	0.302
FEV_1_/FVC ratio(%)	72.13% (59.01%,81.71%)	64.29% (53.63%,76.17%)	0.061
FEV_1_pred (%)	68.88% (47.5%,90.10%)	70.9% (53%,87.32%)	0.932
BSI	10 (7,11.75)	8.5 (5,11)	0.061
E-FACED score	3 (1,4)	2.5 (1,4)	0.375
Cigarette Smokers	16 (9.5%)	1 (2.6%)	0.286
Comorbidity			
Asthma	10 (6.0%)	5 (13.2%)	0.231
COPD	7 (4.2%)	1 (2.6%))	1.000
Diabetes	5 (3.0%)	2 (5.3%)	0.836
Hypertension	11 (6.5%)	5 (13.2%)	0.299
Nasosinusitis	18 (10.7%)	1 (2.6%)	0.213
Inactive pulmonary tuberculosis	30 (17.9%)	2 (5.3%)	0.091

^a^13 participants who were enrolled in both exacerbation and stable cohort

Data was shown as median (IQR) or number (%). *Abbreviations*: *BMI* body mass index, *FEV1* forced expiratory volume in 1 s, *FVC* forced vital capacity, *FACED* forced expiratory volume in 1 s, age, chronic colonization by *Pseudomonas aeruginosa*, extension and dyspnea, *COPD* chronic obstructive pulmonary disease

### Pathogen detection by CMTs and mNGS

**Fig. 2:**
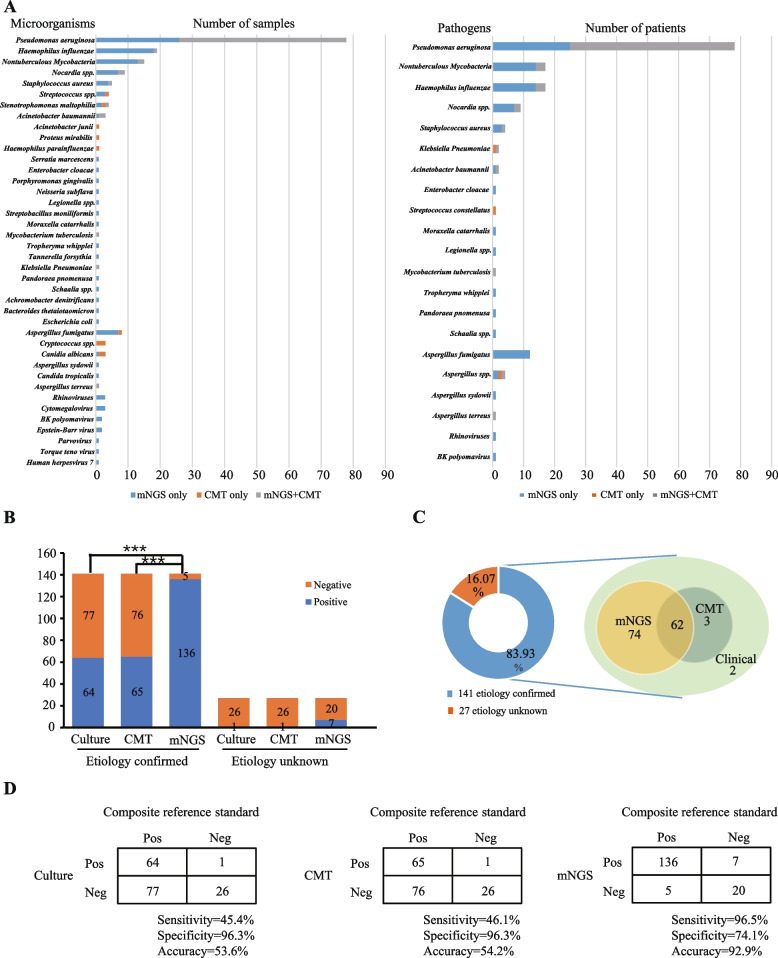
Clinical utility of mNGS for lower airway pathogen identification. **A** Microbial findings in the exacerbation cohort using mNGS, conventional microbiological tests (CMTs), or their combination. The left side represents the number of samples with microorganism detection by mNGS and CMTs, or their combination. The right side represents the number of patients with pathogen detection by mNGS and CMTs, or their combination. **B** Positive and negative rates of pathogen detection by culture, CMTs, and mNGS in the etiology confirmed and unknown groups. **C** Patients’ etiology and diagnosis. The left side shows the percentage of patients with known or unknown etiology, while the right side indicates the number of patients with known etiology diagnosed via mNGS, CMTs, and clinical manifestations. **D** Comparison of culture, CMTs, and mNGS relative to a composite reference standard (CRS). Sensitivity, specificity, and accuracy values are displayed below each table

Potential pathogens were detected via both CMTs and mNGS. 83.9% (141/168) of patients at exacerbation onset were diagnosed as having an infectious etiology according to a composite reference standard. Among these, 96.5% (136/141) of mNGS reports were confirmed by clinical diagnosis. Both CMTs and mNGS could identify bacterial and fungal pathogens, but mNGS showed a higher detection sensitivity for *Pseudomonas aeruginosa*, non-tuberculous mycobacteria, *Haemophilus influenzae*, *Nocardia* spp, and *Staphylococcus aureus*—all were the top 5 pathogens. In addition, mNGS was more sensitive than CMTs for detecting uncommon respiratory pathogens. For example, mNGS had a 100% detection rate for fastidious (*i.e., Legionella* spp., *Neisseria subflava*) and anaerobic bacteria (*i.e.*, *Porphyromonas gingivalis*), but CMTs did not (Fig. 2A). mNGS also showed higher detection rate than CMTs for *Aspergillus fumigatus* (87.5% vs. 12.5%) and cytomegalovirus (100% vs. 0%) which were one of the most frequently detected fungal and viral pathogens, respectively, in our cohort (Fig. 2A).

For patients in the exacerbation cohort with a confirmed infectious etiology, the detection rate was 96.45% for mNGS, which was significantly higher compared with that of culture (45.39%) and CMTs (46.10%, including culture and PCR, G/GM test) (*p* < 0.01, Fig. 2B). mNGS provided additional evidence for infectious etiology in 136 patients. Of these, mNGS alone successfully helped diagnose the infectious etiology in 74 cases, whereas CMTs did not. In contrast, CMTs provided additional evidence for infectious etiology among 65 patients, of whom only 3 had negative mNGS results (Fig. 2C). By comparing with the composite reference standard, the sensitivity of the mNGS was 96.5% (95% CI [91.49%-98.69%]), which was higher than culture (45.4% [37.1%-54.0%], *p* < 0.05) and CMTs (46.1%, [37.7%-54.7%], *p* < 0.05). The specificity of the mNGS assay was 74.1% (95% CI [53.4%-88.1%]), which was lower than culture and CMTs (96.3%, [79.1%-99.8%]). The accuracy of culture, CMTs, and mNGS were 53.6%, 54.2% and 92.9%, respectively (Fig. 2D).

### Correlation of pathogen spectrum with disease severity

Accumulating evidence has shown that infections (including *Pseudomonas aeruginosa* and *Haemophilus influenza*) are associated with disease severity of bronchiectasis [22]. Consistently, we found significant associations between the most frequently detected pathogens in BALF and the clinical characteristics at exacerbation onset. These clinical characteristics included sex, age, body-mass index, duration of disease, radiological severity, BSI, E-FACED score, pulmonary function, smoking, and underlying comorbidities such as asthma, COPD and diabetes. *Pseudomonas aeruginosa* was detected more frequently in patients with longer disease duration (*p* < 0.001) and greater disease severity evidenced by the number of lobes affected, BSI, E-FACED score and the FEV_1_/FVC ratio (Table 2). Notably, non-smokers were more likely to have *Pseudomonas aeruginosa* colonization or infection than smokers (50.0% vs. 12.5%, *p* = 0.004).

**Table 2:** Patient characteristics with and without *Pseudomonas aeruginosa*

			*Pseudomonas aeruginosa*		
		Total number ( *n* = 168)	Yes (*n* = 78)	No ( *n* = 90)	*P* value
Age (years)	/	54 (44,64)	55.5 (45.75,64)	52.5 (44,63.25)	0.489
Sex	Male	46	16	30	0.064
	Female	122	62	60	
BMI	/	20.42 (18.56,22.83)	20.11 (18.24,22.06)	20.80 (18.62,23.61)	0.151
Disease duration (Years)	< 0.5	19	1	18	< 0.001
	0.5—1	8	3	5	
	1—5	26	7	19	
	5—10	54	31	23	
	≥ 10	40	22	18	
Number of lobes affected	1	19	2	17	< 0.001
	2	31	8	23	
	3	99	59	40	
	4	16	8	8	
	5	3	1	2	
FEV_1_ (L)	/	1.60 (1.13,2.09)	1.29 (1.01,1.90)	1.86 (1.18,2.40)	0.012
FEV_1_/FVC	/	72.13% (59.01%,81.71%)	67.70% (56.51%,78.52%)	76.38% (65.80%,85.47%)	0.029
FEV_1_pred (%)	/	68.88% (47.50%,90.10%)	65.40% (41.60%,88.10%)	79.60% (52.60%,94.00%)	0.075
BSI	Mild	2	1	1	< 0.001
	Moderate	59	14	45	
	Severe	107	63	44	
E-FACED score	Mild	118	42	76	< 0.001
	Moderate	50	36	14	
Smoking	/	16	2	14	0.004
Asthma	/	10	4	6	0.675
COPD	/	7	3	4	0.847
Diabetes	/	5	2	3	0.770
Hypertension	/	11	3	8	0.189
Nasosinusitis	/	18	12	6	0.069
Prior tuberculosis	/	30	13	17	0.708

Furthermore, *Haemophilus influenzae* detection was associated with a milder disease severity. 50%, 18.64%, and 5.61% of patients with mild, moderate and severe bronchiectasis, as stratified by the BSI, had *Haemophilus influenzae* detection (*p* = 0.014). The detection rate of *Haemophilus influenzae* was significantly lower in patients with a history of asthma than in those without (40% vs. 9.5%, *p* = 0.003). There were no significant differences for other clinical indices between *Haemophilus influenzae* and non-*Haemophilus influenzae* group (Table 3).

**Table 3:** Patient characteristics with and without *Haemophilus influenzae*

			*Haemophilus influenzae*		
		Total number ( *n* = 168)	Yes (*n* = 19)	No ( *n* = 149)	*P* value
Age (years)	/	54 (44,64)	47 (31,59)	54 (45.5,64)	0.097
Sex	Male	46	4	42	0.513
	Female	122	15	107	
BMI	/	20.42 (18.56,22.83)	20.63 (18.9,23.05)	20.41 (18.34,22.8)	0.345
Disease duration (Years)	< 0.5	19	3	16	0.957
	0.5—1	8	1	7	
	1—5	26	3	23	
	5—10	54	8	46	
	≥ 10	40	4	36	
Number of lobes affected	1	19	3	16	0.498
	2	31	5	26	
	3	99	8	91	
	4	16	3	13	
	5	3	0	3	
FEV_1_ (L)	/	1.60 (1.13,2.09)	2.06 (1.21,2.43)	1.55 (1.05,2.00)	0.211
FEV_1_/FVC	/	72.13% (59.01%,81.71%)	77.27% (58.80%,86.22%)	70.37% (58.50%,81.45%)	0.532
FEV_1_pred (%)	/	68.88% (47.50%,90.10%)	83.60% (50.80%,94.40%)	67.45% (47.28%,90.08%)	0.335
BSI	Mild	2	1	1	0.014
	Moderate	59	11	48	
	Severe	107	7	100	
E-FACED score	Mild	118	16	102	0.158
	Moderate	50	3	47	
Smoking	/	16	3	13	0.325
Asthma	/	10	4	6	0.003
COPD	/	7	2	5	0.142
Diabetes	/	5	0	5	0.419
Hypertension	/	11	0	11	0.222
Nasosinusitis	/	18	3	15	0.449
Prior tuberculosis	/	30	2	28	0.377

We also found a higher detection rate of NTM in patients with a history of inactive pulmonary tuberculosis than those without (20% vs. 6.52%, *p* = 0.019). Intriguingly, bronchiectasis patients without NTM showed poorer lung function evidenced by lower FEV_1_pred (%) (*p* = 0.028). As for other clinical parameters, no statistical differences between NTM and non-NTM group were observed (Table 4).

**Table 4:** Patient characteristics with and without Nontuberculous mycobacteria (NTM)

		Total number ( *n* = 168)	Nontuberculous mycobacteria (NTM)		
			Yes (*n* = 15)	No ( *n* = 153)	*P* value
Age (years)	/	54 (44,64)	55.00 (49.00,66.00)	53.00 (42.50,63.50)	0.168
Sex	Male	46	4	42	0.948
	Female	122	11	111	
BMI	/	20.42 (18.56,22.83)	19.50 (17.65,21.09)	20.55 (18.62,23.12)	0.099
Disease duration (Years)	< 0.5	19	2	17	0.099
	0.5—1	8	3	5	
	1—5	26	2	24	
	5—10	54	4	50	
	≥ 10	40	3	37	
Number of lobes affected	1	19	0	19	0.156
	2	31	1	30	
	3	99	12	87	
	4	16	1	15	
	5	3	1	2	
FEV_1_ (L)	/	1.60 (1.13,2.09)	1.90 (1.57,2.13)	1.48 (1.04,2.10)	0.193
FEV_1_/FVC	/	72.13% (59.01%,81.71%)	74.60% (71.46%,92.36%)	69.26% (57.36%,81.50%)	0.088
FEV_1_pred (%)	/	68.88% (47.50%,90.10%)	88.10 (79.25%,96.65%)	66.60% (47.08%,90.00%)	0.028
BSI	Mild	2	0	2	0.681
	Moderate	59	4	55	
	Severe	107	11	96	
E-FACED score	Mild	118	13	105	0.146
	Moderate	50	2	48	
Smoking	/	16	2	14	0.600
Asthma	/	10	1	9	0.903
COPD	/	7	0	7	0.399
Diabetes	/	5	0	5	0.479
Hypertension	/	11	1	10	0.984
Nasosinusitis	/	18	1	17	0.596
Prior tuberculosis	/	30	6	24	0.019

### Higher API of PPMs correlated with a shorter time to the next exacerbation

**Fig. 3:**
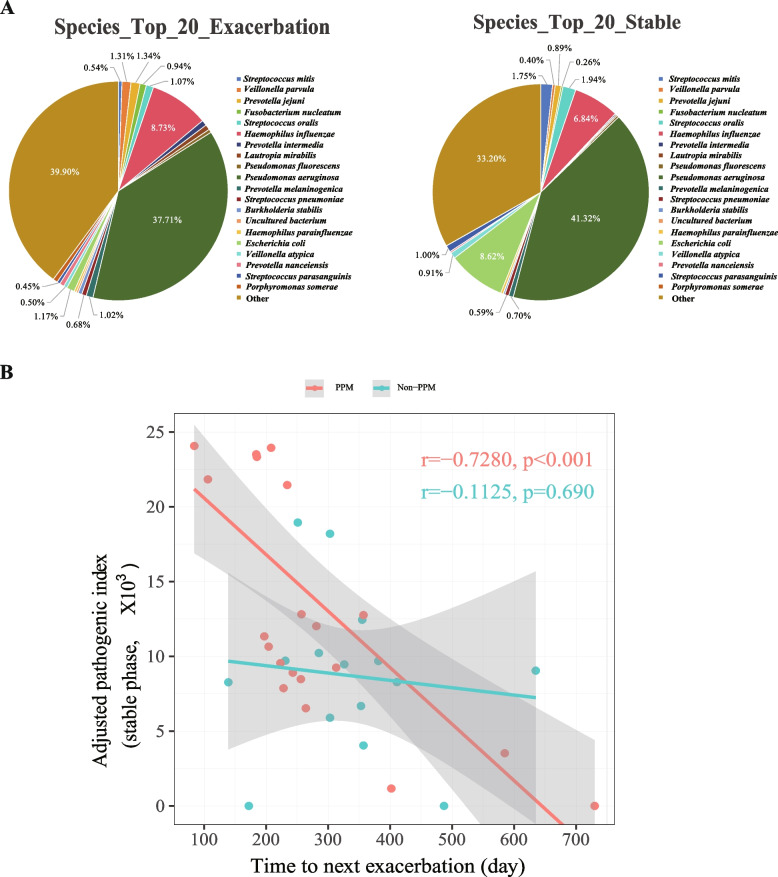
The pathogenic index of potential pathogenic microorganisms (PPMs) negatively correlates with the time to next exacerbation. **A** The composition of top 20 bacterial species measured by mNGS in the lower airways in bronchiectasis patients at exacerbation onset and stable state. **B** Pearson’s analysis of correlation of adjusted pathogenic index (API) with the time to next exacerbation in bronchiectasis patients with or without PPMs colonization

We firstly analyzed the major microbial composition in BALF from bronchiectasis patients, and observed a similar microbial composition, while bacterial abundance of each species was different between exacerbation onset and stable state (Fig. 3A). Previous studies have shown that colonization of PPMs was associated with increased inflammation and poorer clinical outcomes of chronic lung diseases such as COPD [7, 23]. Similarly, we found that higher API was significantly associated with shorter time to next exacerbation in bronchiectasis patients with PPMs colonization, but not in non-PPMs group (Fig. 3B). To mitigate the influence of highly leveraged data points, we performed an outlier exclusion analysis. The correlation remained significant for the PPM group (*r* = -0.7280, *P* < 0.001) but not for the non-PPM group (*r* = -0.3327, *P* = 0.245). These data suggest that both the colonization and bacterial load of PPMs in BALF was associated with poorer clinical outcomes in bronchiectasis.

## Discussion

Infection has been a common trigger of the pathogenesis and exacerbation of bronchiectasis [5]. Identifying the probable or definite pathogens is crucial for personalized treatment for exacerbation. Although being the most common sample that be routine clinical used for pathogen identification in bronchiectasis, sputum remains the suboptimal source because the sputum volume is highly variable and suffers from the contamination of oral and upper airway contents. Therefore, lower respiratory tract samples such as BALF are more ideal samples for pathogen identification, while BALF has low microbial biomass and requires higher technical requirements for the operators. Here, we collected BALF samples to identify microbial characteristics in the lower airways in bronchiectasis via mNGS, which has been widely used to rapidly and precisely detect pathogens in patients with lower respiratory tract infection and bronchiectasis [10, 24]. Consistently, mNGS yielded superior sensitivity, specificity and accuracy compare with CMTs. Previous studies have shown that *Pseudomonas aeruginosa*, *Haemophilus influenzae*, *rhinovirus* and *influenza viruses* are the most common pathogens at exacerbation onset of bronchiectasis [25–27]. In support of these studies, we also found that *Pseudomonas aeruginosa*, *Haemophilus influenza*, and other pathogens were frequently detected in BALF from bronchiectasis patients at exacerbation onset, and that *Pseudomonas aeruginosa* was associated with a longer disease duration. We have also shown that mNGS could be a better diagnostic tool than CTMs in detecting NTM, *Nocardia*, and *Legionella*, indicating that these pathogens may be underrepresented in earlier studies and overlooked clinically. To our knowledge, this is the largest cohort study characterizing the microbial spectrum in the lower airways in bronchiectasis patients via collection of BALF samples.

Accumulating evidence has shown that PPMs colonization impairs efferocytosis of alveolar macrophages and is associated with more rapid decline of lung function and poorer clinical outcomes in COPD [7, 28]. Consistently, PPM colonization in the lower airways was common in bronchiectasis patients, and was associated with a shorter time to the next exacerbation. Therefore, PPM colonization might be linked to disease progression in bronchiectasis. Immune escape of PPMs in the stable state, as reported previously, would predispose to the overgrowth and transition into the pathogenic state, resulting in exacerbation of COPD or other chronic lung diseases [8, 21]. In support of these findings, we found that a higher load of PPMs (referred to as API) in the lower airways was associated with a significantly shorter time to the next exacerbation in bronchiectasis at stable state. Our data imply that the bacterial burden of colonized PPMs in the lower airways was crucial for predicting the course of exacerbation and deterioration in bronchiectasis patients. Meanwhile, our study provides therapeutic clues that long-term antibiotic treatment should be applied to reduce the loads of PPMs among patients with PPM colonization. This strategy has resulted in the reduced frequency of exacerbation and improved quality of life in bronchiectasis patients [29, 30].

Our study has several limitations. First, mNGS provides various useful information and potential pathogenic candidates for pathogens identification, but it remains difficult to distinguish pathogens from colonizing microorganisms. Thus, microbiological and radiological examination and rational adjudication by clinicians are needed to more accurately identify the definite pathogens in bronchiectasis. Additionally, because API is calculated based on the reads of microbial species by mNGS, it is unable to provide an absolute quantification of microbial loads in clinical practice. Therefore, quantitative PCR is necessary to monitor the dynamic changes of bacterial burden of PPMs, which can help to precisely dissect the relationship of PPMs with the disease severity in real-world. Moreover, accurately pinpointing disease duration remains a great challenge in bronchiectasis, as symptoms often appear later in the progression. Although our data showed a positive correlation between disease duration (based on first radiological diagnosis) and colonization by *Pseudomonas aeruginosa* and NTM, it remains unable to reflect the true onset or exact start of disease. Thus, it is necessary and urgent to establish method for precisely determining disease duration in bronchiectasis.

## Conclusion

We have investigated the pathogen spectrum by combination of mNGS and CMTs in a cohort of bronchiectasis patients with exacerbation onset based on BALF samples. Our study has not only shown more accurate characterization of the pathogenic bacteria responsible for exacerbations, but also provides the evidence for the prognostic role of colonization and load of PPMs in bronchiectasis.

### Supplementary Information

Supplementay Material 1.

## Data Availability

The original contributions presented in the study are publicly available. The sequencing data reported in this study was archived in the Sequence Read Archive (SRA) with the accession number (PRJNA1021363).

## Abbreviations

BALF: Bronchoalveolar lavage fluid
CMTs: Conventional microbiological tests
mNGS: Metagenomic Next-Generation Sequencing
PPMs: Potential pathogenic microorganisms
API: Adjusted pathogenic index
